# Crystal structure of 4-azido­methyl-6-*tert*-butyl-2*H*-chromen-2-one

**DOI:** 10.1107/S205698901500290X

**Published:** 2015-03-04

**Authors:** Nasseem El-Khatatneh, Chandra, D. Shamala, K. Shivashankar, Mukhokosi Emma Panzi, M. Mahendra

**Affiliations:** a Department of Studies in Physics, Manasagangotri, University of Mysore, Mysore 570 006, India; b Department of Chemistry, Central College Campus, Bangalore University, Bangalore 560 001, India

**Keywords:** crystal structure, chromene, coumarin, hydrogen bonding

## Abstract

In the title compound, C_14_H_15_N_3_O_2_, one of the methyl C atoms of the *tert*-butyl group lies almost in the plane of the chromene ring system [deviation = −0.097 (2) Å], one lies above and one lies below [deviations = 1.460 (3) and 1.006 (3) Å, respectively]. The C—C—N—N torsion angle is 142.33 (17)°. In the crystal, moelcules are linked by weak C—H⋯O hydrogen bonds to generate *C*(6) chains propagating in the [010] direction.

## Related literature   

For background to the biological properties of coumarins, see: Basanagouda *et al.* (2009 ▸); Liu *et al.* (2008 ▸); Mustafa *et al.* (2011 ▸); Ronad *et al.* (2008 ▸); Tian *et al.* (2000 ▸); Puttaraju *et al.* (2013 ▸). For a related structure, see: Chandra *et al.* (2014 ▸).
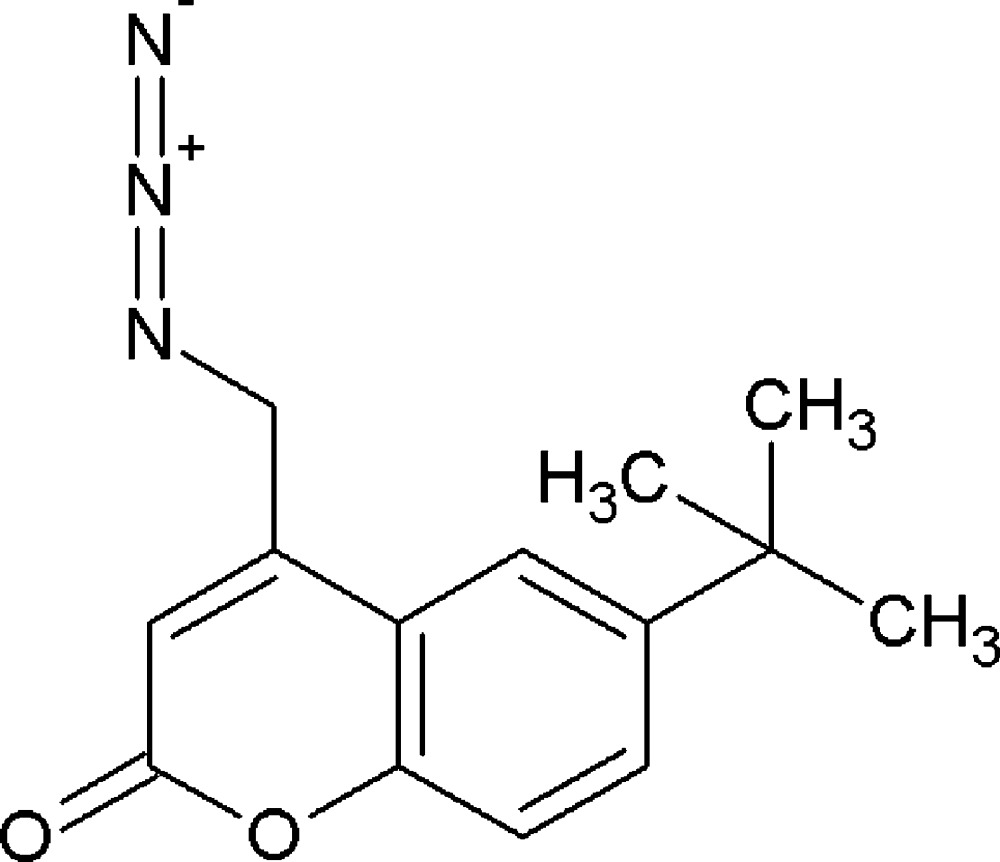


## Experimental   

### Crystal data  

C_14_H_15_N_3_O_2_
*M* *_r_* = 257.29
Monoclinic, 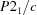

*a* = 10.6816 (7) Å
*b* = 11.1416 (8) Å
*c* = 11.5409 (8) Å
β = 100.674 (4)°
*V* = 1349.72 (16) Å^3^
*Z* = 4
Cu *K*α radiation
μ = 0.71 mm^−1^
*T* = 293 K
0.30 × 0.25 × 0.20 mm

### Data collection  

Bruker X8 Proteum diffractometer
5911 measured reflections
2165 independent reflections
1949 reflections with *I* > 2σ(*I*)
*R*_int_ = 0.037

### Refinement  

*R*[*F* ^2^ > 2σ(*F* ^2^)] = 0.045
*wR*(*F* ^2^) = 0.132
*S* = 1.04
2165 reflections
175 parameters
H-atom parameters constrained
Δρ_max_ = 0.13 e Å^−3^
Δρ_min_ = −0.17 e Å^−3^

### 

Data collection: *APEX2* (Bruker, 2009 ▸); cell refinement: *SAINT* (Bruker, 2009 ▸); data reduction: *SAINT*; program(s) used to solve structure: *SHELXS97* (Sheldrick, 2008 ▸); program(s) used to refine structure: *SHELXL97* (Sheldrick, 2008 ▸); molecular graphics: *PLATON* (Spek, 2009 ▸); software used to prepare material for publication: *SHELXL97*.

## Supplementary Material

Crystal structure: contains datablock(s) global, I. DOI: 10.1107/S205698901500290X/hb7363sup1.cif

Structure factors: contains datablock(s) I. DOI: 10.1107/S205698901500290X/hb7363Isup2.hkl

Supporting information file. DOI: 10.1107/S205698901500290X/hb7363Isup3.cml

**Fig. 1.**
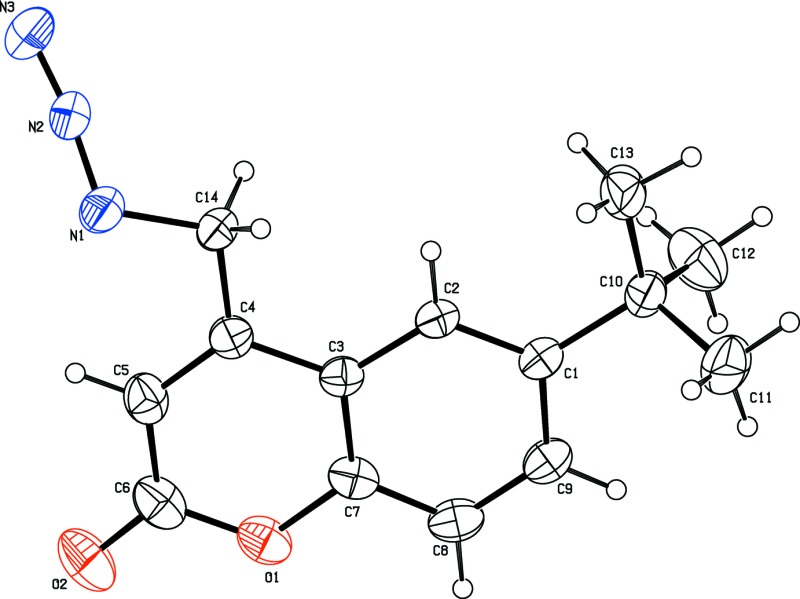
Perspective diagram of the molecule with 50% probability displacement ellipsoids.

**Fig. 2.**
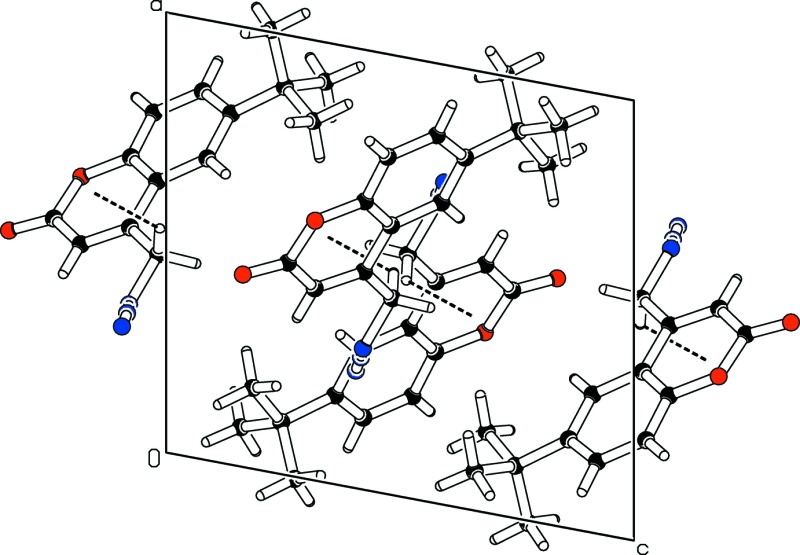
Packing diagram of the molecule viewed parallel to the b axis.

. DOI: 10.1107/S205698901500290X/hb7363fig1.tif
Perspective diagram of the mol­ecule with 50% probability displacement ellipsoids.

b . DOI: 10.1107/S205698901500290X/hb7363fig2.tif
Packing diagram of the mol­ecule viewed parallel to the *b* axis.

CCDC reference: 1048730

Additional supporting information:  crystallographic information; 3D view; checkCIF report

## Figures and Tables

**Table 1:** Hydrogen-bond geometry (, )

*D*H*A*	*D*H	H*A*	*D* *A*	*D*H*A*
C14H14*A*O2^i^	0.97	2.55	3.311(2)	135

Symmetry code: (i) 
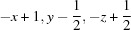
.

## supplementary crystallographic information

### S1. Comment

Coumarin and its substituents are of well known heterocyclic compounds, which have a variety of biologically activities; such as anti-tumour (Mustafa *et al.*, 2011), anti-bacterial (Basanagouda *et al.*, 2009; Liu *et al.*, 2008) and analgesic (Ronad *et al.*, 2008) agents. In addition, coumarin derivatives have been found to be very useful in many applications; such as nonlinear optical materials and as intermediates for the drug synthesis (Tian *et al.*, 2000). In our previous work (Puttaraju *et al.*, 2013), we have reported the synthesis, *in vitro* antimicrobial and anticancer activities of new coumarin derivatives substituted dihydrobenzo[4,5]imidazo[1,2-*a*]pyrimidin-4-ones. In continuation to this, we have synthesized the title compound to study its molecular and crystal structure.

In the molecular structure of the title compound (Fig. 1), the chromene moiety is almost planar, with the maximum deviation from the mean plane being 0.093 (2) Å for atom C10, respectively. The azidomethyl group is in *anti-periplanar* conformation with respect to the chromene moiety, as indicated by the torsion angle value of 172.35 (14)° (C3–C4–C14–N1). The bond lengths and angles are within normal ranges and are comparable to related structure (Chandra *et al.*, 2014). The crystal structure features C—H···O hydrogen bonds, which link the molecules into [010] chains, as shown in Fig. 2.

### S2. Experimental

6-*tert*-Butyl-4-bromomethylcoumarins (0.001 mmol. 0.5 g) were taken in 15 ml acetone in a round bottomed flask and stirred. To this, sodium azide (0.002 mol, 0.13 g) in 5 ml of water was added drop wise with stirring, which was continued for 3 hrs (reaction was monitored by TLC). The reaction mixture was poured in to ice cold water, separated solid was filtered and recrystallized from ethyl alcohol to get pale yelllow blocks of the title compound.

### S3. Refinement

The H atoms were positioned geometrically and allowed to ride on their parent atom, with C–H distance in the range of 0.93 to 0.97 Å; *U*_iso_(H) = 1.2–1.5*U*_eq_ (carrier atom) for all H atoms.

### Crystal data

**Table:**

C_14_H_15_N_3_O_2_	*F*(000) = 544
*M**_r_* = 257.29	*D*_x_ = 1.266 Mg m^−^^3^
Monoclinic, *P*2_1_/*c*	Cu *K*α radiation, λ = 1.54178 Å
Hall symbol: -P 2ybc	Cell parameters from 2165 reflections
*a* = 10.6816 (7) Å	θ = 5.6–64.5°
*b* = 11.1416 (8) Å	µ = 0.71 mm^−^^1^
*c* = 11.5409 (8) Å	*T* = 293 K
β = 100.674 (4)°	Block, pale yellow
*V* = 1349.72 (16) Å^3^	0.30 × 0.25 × 0.20 mm
*Z* = 4	

### Data collection

**Table:**

Bruker X8 Proteum diffractometer	1949 reflections with *I* > 2σ(*I*)
Radiation source: Bruker MicroStar microfocus rotating anode	*R*_int_ = 0.037
Helios multilayer optics monochromator	θ_max_ = 64.5°, θ_min_ = 5.6°
Detector resolution: 10.7 pixels mm^-1^	*h* = −12→12
φ and ω scans	*k* = −12→12
5911 measured reflections	*l* = −13→13
2165 independent reflections	

### Refinement

**Table:**

Refinement on *F*^2^	Primary atom site location: structure-invariant direct methods
Least-squares matrix: full	Secondary atom site location: difference Fourier map
*R*[*F*^2^ > 2σ(*F*^2^)] = 0.045	Hydrogen site location: inferred from neighbouring sites
*wR*(*F*^2^) = 0.132	H-atom parameters constrained
*S* = 1.04	*w* = 1/[σ^2^(*F*_o_^2^) + (0.0667*P*)^2^ + 0.2532*P*] where *P* = (*F*_o_^2^ + 2*F*_c_^2^)/3
2165 reflections	(Δ/σ)_max_ < 0.001
175 parameters	Δρ_max_ = 0.13 e Å^−^^3^
0 restraints	Δρ_min_ = −0.17 e Å^−^^3^

### Special details

**Table:**

Geometry. Bond distances, angles *etc*. have been calculated using the rounded fractional coordinates. All su's are estimated from the variances of the (full) variance-covariance matrix. The cell e.s.d.'s are taken into account in the estimation of distances, angles and torsion angles
Refinement. Refinement on *F*^2^ for ALL reflections except those flagged by the user for potential systematic errors. Weighted *R*-factors *wR* and all goodnesses of fit *S* are based on *F*^2^, conventional *R*-factors *R* are based on *F*, with *F* set to zero for negative *F*^2^. The observed criterion of *F*^2^ > σ(*F*^2^) is used only for calculating -*R*-factor-obs *etc*. and is not relevant to the choice of reflections for refinement. *R*-factors based on *F*^2^ are statistically about twice as large as those based on *F*, and *R*-factors based on ALL data will be even larger.

### Fractional atomic coordinates and isotropic or equivalent isotropic displacement parameters (Å^2^)

**Table:**

	*x*	*y*	*z*	*U*_iso_*/*U*_eq_	
O1	0.59056 (12)	0.02266 (11)	0.31873 (10)	0.0633 (4)	
O2	0.43714 (15)	0.00517 (14)	0.16423 (12)	0.0886 (6)	
N1	0.32064 (15)	−0.31631 (13)	0.42079 (19)	0.0852 (7)	
N2	0.29968 (12)	−0.42384 (13)	0.41655 (12)	0.0581 (5)	
N3	0.26741 (17)	−0.51960 (15)	0.40562 (16)	0.0784 (7)	
C1	0.79791 (14)	−0.09815 (13)	0.63907 (13)	0.0464 (5)	
C2	0.68585 (13)	−0.15469 (12)	0.58820 (12)	0.0441 (4)	
C3	0.61248 (13)	−0.11775 (12)	0.48071 (12)	0.0429 (4)	
C4	0.49582 (14)	−0.17576 (13)	0.42299 (13)	0.0478 (5)	
C5	0.43662 (16)	−0.13424 (15)	0.31759 (14)	0.0585 (6)	
C6	0.48347 (19)	−0.03397 (17)	0.26019 (15)	0.0642 (6)	
C7	0.65570 (15)	−0.01975 (13)	0.42495 (13)	0.0493 (5)	
C8	0.76681 (17)	0.03930 (15)	0.47367 (16)	0.0593 (6)	
C9	0.83629 (16)	−0.00013 (14)	0.57861 (16)	0.0566 (5)	
C10	0.88114 (15)	−0.14115 (14)	0.75389 (14)	0.0553 (5)	
C11	0.9105 (3)	−0.0370 (2)	0.8404 (2)	0.0925 (9)	
C12	1.0051 (2)	−0.1916 (3)	0.7259 (2)	0.0969 (10)	
C13	0.8160 (2)	−0.2375 (2)	0.81437 (18)	0.0840 (8)	
C14	0.44697 (15)	−0.27965 (14)	0.48332 (16)	0.0581 (5)	
H2	0.65810	−0.21990	0.62690	0.0530*	
H5	0.36230	−0.17200	0.28040	0.0700*	
H8	0.79420	0.10520	0.43560	0.0710*	
H9	0.91130	0.03960	0.61060	0.0680*	
H11A	0.83230	−0.00230	0.85410	0.1390*	
H11B	0.95900	0.02280	0.80810	0.1390*	
H11C	0.95890	−0.06580	0.91360	0.1390*	
H12A	1.05700	−0.22160	0.79690	0.1450*	
H12B	1.05020	−0.12930	0.69340	0.1450*	
H12C	0.98610	−0.25570	0.66980	0.1450*	
H13A	0.73560	−0.20790	0.82820	0.1260*	
H13B	0.86890	−0.25830	0.88820	0.1260*	
H13C	0.80210	−0.30740	0.76490	0.1260*	
H14A	0.50560	−0.34650	0.48640	0.0700*	
H14B	0.44200	−0.25750	0.56360	0.0700*	

### Atomic displacement parameters (Å^2^)

**Table:**

	*U*^11^	*U*^22^	*U*^33^	*U*^12^	*U*^13^	*U*^23^
O1	0.0776 (8)	0.0686 (8)	0.0471 (7)	0.0085 (6)	0.0204 (6)	0.0127 (5)
O2	0.1016 (11)	0.1144 (12)	0.0476 (8)	0.0252 (9)	0.0082 (7)	0.0190 (7)
N1	0.0611 (9)	0.0489 (9)	0.1292 (15)	−0.0037 (7)	−0.0249 (9)	−0.0015 (8)
N2	0.0521 (8)	0.0577 (9)	0.0598 (9)	−0.0068 (6)	−0.0019 (6)	−0.0048 (6)
N3	0.0802 (11)	0.0653 (11)	0.0841 (12)	−0.0237 (8)	0.0010 (9)	−0.0092 (8)
C1	0.0489 (8)	0.0428 (8)	0.0493 (8)	−0.0053 (6)	0.0141 (6)	−0.0055 (6)
C2	0.0509 (8)	0.0375 (7)	0.0453 (8)	−0.0036 (6)	0.0128 (6)	−0.0017 (6)
C3	0.0502 (8)	0.0387 (7)	0.0419 (8)	0.0032 (6)	0.0143 (6)	−0.0056 (6)
C4	0.0539 (8)	0.0422 (8)	0.0467 (8)	0.0079 (6)	0.0080 (6)	−0.0100 (6)
C5	0.0616 (10)	0.0630 (10)	0.0482 (9)	0.0110 (8)	0.0030 (7)	−0.0106 (7)
C6	0.0770 (12)	0.0753 (11)	0.0424 (9)	0.0209 (9)	0.0166 (8)	0.0016 (8)
C7	0.0617 (9)	0.0488 (8)	0.0422 (8)	0.0082 (7)	0.0223 (7)	0.0029 (6)
C8	0.0679 (10)	0.0516 (9)	0.0653 (11)	−0.0080 (8)	0.0307 (8)	0.0070 (7)
C9	0.0556 (9)	0.0527 (9)	0.0644 (10)	−0.0122 (7)	0.0189 (8)	−0.0018 (7)
C10	0.0535 (9)	0.0556 (9)	0.0541 (9)	−0.0116 (7)	0.0027 (7)	−0.0021 (7)
C11	0.1146 (18)	0.0844 (14)	0.0685 (13)	−0.0236 (13)	−0.0093 (12)	−0.0150 (11)
C12	0.0754 (13)	0.1139 (19)	0.0993 (17)	0.0241 (12)	0.0111 (12)	0.0184 (14)
C13	0.0873 (13)	0.0933 (15)	0.0614 (11)	−0.0298 (11)	−0.0126 (10)	0.0242 (10)
C14	0.0529 (9)	0.0432 (8)	0.0715 (10)	−0.0048 (7)	−0.0059 (7)	−0.0042 (7)

### Geometric parameters (Å, º)

**Table:**

O1—C6	1.370 (2)	C10—C12	1.527 (3)
O1—C7	1.3763 (19)	C10—C13	1.518 (3)
O2—C6	1.208 (2)	C2—H2	0.9300
N1—N2	1.218 (2)	C5—H5	0.9300
N1—C14	1.466 (2)	C8—H8	0.9300
N2—N3	1.121 (2)	C9—H9	0.9300
C1—C2	1.384 (2)	C11—H11A	0.9600
C1—C9	1.398 (2)	C11—H11B	0.9600
C1—C10	1.530 (2)	C11—H11C	0.9600
C2—C3	1.4008 (19)	C12—H12A	0.9600
C3—C4	1.452 (2)	C12—H12B	0.9600
C3—C7	1.389 (2)	C12—H12C	0.9600
C4—C5	1.345 (2)	C13—H13A	0.9600
C4—C14	1.494 (2)	C13—H13B	0.9600
C5—C6	1.435 (3)	C13—H13C	0.9600
C7—C8	1.382 (2)	C14—H14A	0.9700
C8—C9	1.370 (3)	C14—H14B	0.9700
C10—C11	1.525 (3)		
			
C6—O1—C7	121.29 (13)	C3—C2—H2	119.00
N2—N1—C14	116.07 (15)	C4—C5—H5	119.00
N1—N2—N3	172.26 (18)	C6—C5—H5	119.00
C2—C1—C9	117.04 (14)	C7—C8—H8	120.00
C2—C1—C10	122.92 (13)	C9—C8—H8	120.00
C9—C1—C10	120.01 (14)	C1—C9—H9	119.00
C1—C2—C3	122.70 (13)	C8—C9—H9	119.00
C2—C3—C4	124.58 (13)	C10—C11—H11A	110.00
C2—C3—C7	117.50 (13)	C10—C11—H11B	110.00
C4—C3—C7	117.91 (13)	C10—C11—H11C	109.00
C3—C4—C5	118.81 (14)	H11A—C11—H11B	109.00
C3—C4—C14	118.39 (13)	H11A—C11—H11C	109.00
C5—C4—C14	122.81 (15)	H11B—C11—H11C	109.00
C4—C5—C6	122.65 (16)	C10—C12—H12A	109.00
O1—C6—O2	116.67 (17)	C10—C12—H12B	110.00
O1—C6—C5	117.49 (15)	C10—C12—H12C	109.00
O2—C6—C5	125.85 (18)	H12A—C12—H12B	109.00
O1—C7—C3	121.73 (14)	H12A—C12—H12C	109.00
O1—C7—C8	116.95 (14)	H12B—C12—H12C	109.00
C3—C7—C8	121.31 (14)	C10—C13—H13A	109.00
C7—C8—C9	119.38 (15)	C10—C13—H13B	110.00
C1—C9—C8	122.06 (16)	C10—C13—H13C	109.00
C1—C10—C11	110.18 (14)	H13A—C13—H13B	109.00
C1—C10—C12	108.64 (14)	H13A—C13—H13C	109.00
C1—C10—C13	112.18 (14)	H13B—C13—H13C	109.00
C11—C10—C12	109.72 (19)	N1—C14—H14A	109.00
C11—C10—C13	107.06 (16)	N1—C14—H14B	109.00
C12—C10—C13	109.04 (17)	C4—C14—H14A	109.00
N1—C14—C4	110.84 (14)	C4—C14—H14B	109.00
C1—C2—H2	119.00	H14A—C14—H14B	108.00
			
C7—O1—C6—O2	−175.54 (16)	C2—C3—C4—C5	−177.54 (15)
C7—O1—C6—C5	4.2 (2)	C2—C3—C4—C14	2.2 (2)
C6—O1—C7—C3	−3.5 (2)	C7—C3—C4—C14	−178.75 (14)
C6—O1—C7—C8	176.23 (16)	C2—C3—C7—O1	179.59 (13)
N2—N1—C14—C4	142.33 (17)	C2—C3—C7—C8	−0.1 (2)
C10—C1—C2—C3	−177.49 (14)	C4—C3—C7—O1	0.5 (2)
C2—C1—C9—C8	0.1 (2)	C4—C3—C7—C8	−179.21 (15)
C10—C1—C9—C8	177.99 (15)	C7—C3—C4—C5	1.5 (2)
C2—C1—C10—C11	−128.80 (18)	C3—C4—C5—C6	−0.6 (2)
C2—C1—C10—C12	110.99 (19)	C14—C4—C5—C6	179.65 (16)
C2—C1—C10—C13	−9.6 (2)	C5—C4—C14—N1	−7.9 (2)
C9—C1—C2—C3	0.3 (2)	C3—C4—C14—N1	172.35 (14)
C9—C1—C10—C12	−66.8 (2)	C4—C5—C6—O1	−2.2 (3)
C9—C1—C10—C13	172.62 (15)	C4—C5—C6—O2	177.53 (19)
C9—C1—C10—C11	53.5 (2)	C3—C7—C8—C9	0.5 (2)
C1—C2—C3—C4	178.72 (14)	O1—C7—C8—C9	−179.18 (15)
C1—C2—C3—C7	−0.3 (2)	C7—C8—C9—C1	−0.5 (3)

### Hydrogen-bond geometry (Å, º)

**Table:**

*D*—H···*A*	*D*—H	H···*A*	*D*···*A*	*D*—H···*A*
C14—H14*A*···O2^i^	0.97	2.55	3.311 (2)	135

Symmetry code: (i) −*x*+1, *y*−1/2, −*z*+1/2.
