# Supplementary figures and images for: Crystal structure of 4-azido­methyl-6-tert-butyl-2H-chromen-2-one
Source: Acta Crystallogr E Crystallogr Commun. 2015 Mar 4;71(Pt 4):o218–9. doi: 10.1107/S205698901500290X (PMC4438804; doi:10.1107/S205698901500290X)

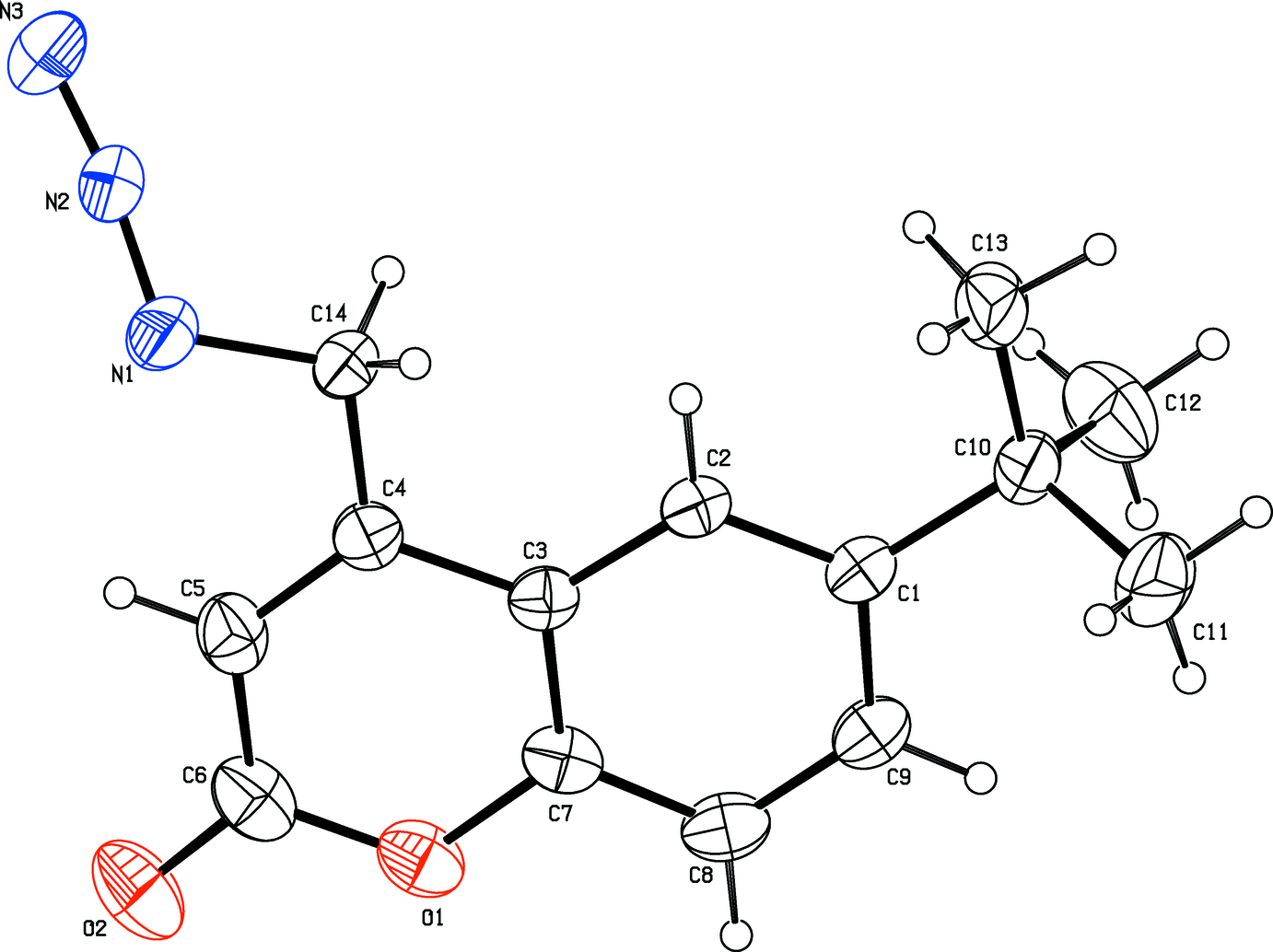

Supplement: Supplementary file 4 [file e-71-0o218-fig1.tif]

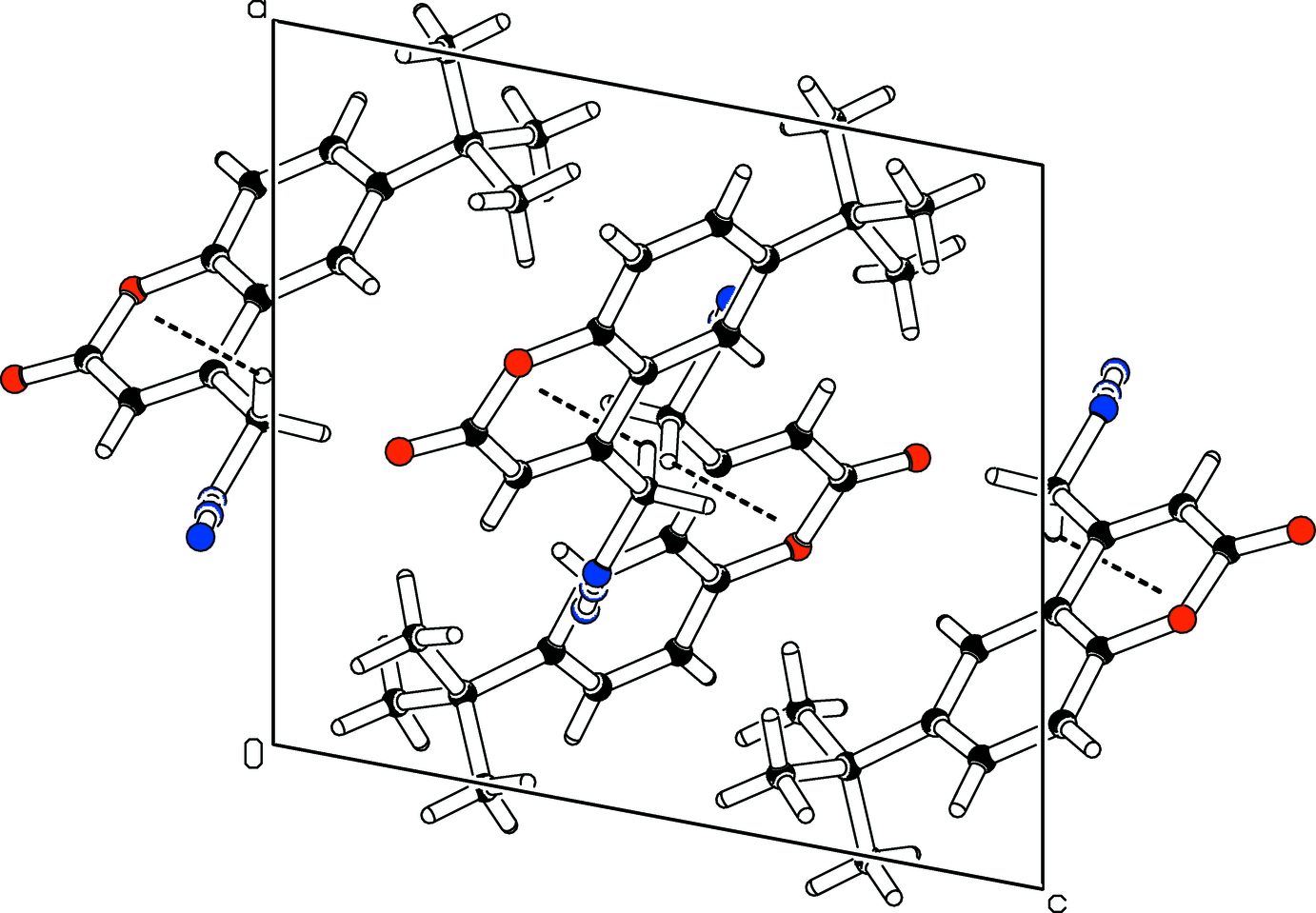

Supplement: Supplementary file 5 [file e-71-0o218-fig2.tif]
